# Analysis of factors affecting the surgical efficacy for elderly intertrochanteric fracture

**DOI:** 10.3389/fsurg.2025.1589181

**Published:** 2025-04-15

**Authors:** Xiang Yu, Yu-Zhi Li, Hai-Jian Lu, Rong-Guang Ao, Bing-Li Liu

**Affiliations:** Department of Orthopedics, Shanghai Seventh People’s Hospital, Shanghai, China

**Keywords:** intertrochanteric fracture, the elderly, fracture fixation, risk factors, the lateral wall, tip-apex distance

## Abstract

**Objective:**

To explore the relevant factors affecting the efficacy of surgery for intertrochanteric fracture.

**Methods:**

A retrospective case series study was conducted to analyze the clinical data of 212 patients with intertrochanteric fractures from January 2021 to December 2023. The patients, comprising 100 males and 112 females aged 65–98 years old (mean age 77.8 ± 10.5 years), were categorized based on fracture healing outcomes into a normal healing group (163 cases) and an abnormal healing group (49 cases, including 2 cases of non-union). Factors such as gender, age, injury side, fracture classification, thickness of femoral lateral wall, medial support, tip-apex distance and fixation position were recorded and analyzed through Logistic regression to identify the main factors influencing fracture healing.

**Results:**

Univariate analysis revealed statistically significant differences in AO classification, Evans-Jensen classification, medial support, tip-apex distance and main screw position between the two groups (*P* < 0.05). Logistic regression analysis indicated that AO type A1 (OR = 1.030), medial support (OR = 0.395), tip-apex distance ≤25 mm (OR = 0.266) and main screw located in the middle and lower part of the femoral head (OR = 0.986) were significantly related to fracture union (*P* < 0.05). The Oxford score of the normal fracture healing group (mean 42.6 ± 4.5 points) was higher than that of the abnormal healing group (mean 35.4 ± 3.2 points) (*P* < 0.05).

**Conclusions:**

The stability of internal fixation is the most important factor affecting intertrochanteric fracture healing. The medial support, tip-apex distance ≤25 mm and the position of the screw are helpful for fracture healing and recovery of joint function.

## Introduction

Intertrochanteric fracture constitutes approximately 35.7% of hip fracture and 3%–4% of total body fractures, predominantly affecting individuals aged 65 and above (1). Surgical intervention is the primary approach for managing intertrochanteric fracture. However, complications such as fracture nonunion, malunion and internal fixation failure may arise due to factors like fracture displacement, bone density, and fixation stability (2). Fracture healing can be influenced by variables such as fracture type, bone density, extent of fracture reduction, choice of internal fixation, and placement of the fixation (3). Clinicians can control the latter three factors. Treatment decisions should be guided by the first two factors, with emphasis on selecting appropriate fixation methods and optimal positioning to achieve maximal stability, facilitate fracture healing, and prevent complications.

The lateral wall, extending from the vastus lateralis muscle origin to the lesser trochanter plane in the proximal femur, is a crucial factor in assessing intertrochanteric fracture stability and predicting healing (4, 5). It offers lateral support to head and neck bone fragments during surgery, effectively countering medial shaft shift, fragment rotation, varus deformity, and screw loosening. Tip-apex distance(TAD) is a key parameter for assessing internal fixation position on the femoral head and predicting screw penetration (6). This study combines the AO classification with the Evans-Jensen classification for the first time to analyze the stability of internal fixation, we retrospectively analyzed clinical data from 212 intertrochanteric fracture patients between January 2021 to December 2023. The aim was to identify factors influencing clinical outcomes and provide insights for enhancing treatment efficacy and standardizing procedures.

## Materials and methods

### Normal information

Inclusion criteria: (1) Intertrochanteric fracture (AO31-A1/A2). (2) Be older than 65 years. (3) Time from injury to treatment <48 hours; (4) Clear history of trauma. (5) No other fractures History. (6) Treatment with closed/open reduction and internal fixation using Dynamic Hip Screws (DHS), Proximal Femoral Nail Antirotation (PFNA), InterTAN Hip Fracture Nailing System(InterTan) or Proximal Femoral Locking Plate (PFLP). Exclusion criteria: (1) Pathological fracture. (2) Lost to follow-up. (3) Incomplete clinical data.

A total of 420 cases from January 2021 to December 2023 were identified. After excluding 124 cases that did not follow up as planned and 84 cases with incomplete imaging data, we included 212 patients with intertrochanteric fractures who met the specified criteria, comprising 100 males and 112 females with an age range of 65–98 years [(77.8 ± 10.5) years old]. The causes of injury were falling on level ground (186 cases), traffic accidents (14 cases), falls from height (8 cases), and other causes (4 cases). Among the patients, 163 experienced normal fracture healing (normal time and neck-shaft angle difference ≤5°) (normal healing group), while 49 cases showed abnormal healing patterns such as delayed healing, non-healing, or neck-shaft angle difference >5° (abnormal healing group）. Additionally, there was 2 cases of fracture non-union. Fractures were classified based on AO type (55 cases A1, 157 cases A2) and Evans-Jensen classification (17 cases type I, 37 cases type II, 75 cases type III, 58 cases type IV, 25 cases type V). The lateral wall thickness was categorized as less than 10 mm (94 cases), 10–20 mm (67 cases), 20–30 mm (38 cases), and more than 30 mm (13 cases). The presence of defects in the medial wall was noted as partial defect (78 cases), complete defect (77 cases), and no defect (57 cases). The time from injury to surgery ranged from 2 to 11 days, with an average of 5 days.

### Surgical method

**Figure 1:**
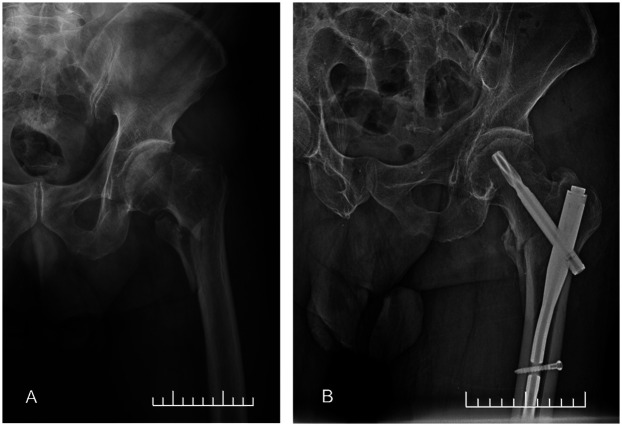
Internal fixation of intertrochanteric fracture. **(A)** Preoperative frontal x-ray. **(B)** Postoperative frontal x-ray.

All patients were initially assessed in the emergency department and underwent x-rays for confirmation of diagnosis. Treatment included traction, swelling management, and prevention of deep vein thrombosis. In cases where necessary, a three-dimensional CT examination of the joints was conducted. Following this, all patients underwent surgical intervention under general anesthesia. Fractures were closed and reduced using a traction bed, with the option of a small incision for assistance in reduction. Internal fixation material was placed after guiding a pin under fluoroscopy. Post-fixation, another fluoroscopy was performed to ensure proper reduction and fixation, followed by suturing of the incision. For relatively stable type A1 fractures, fixation was done using DHS and PFNA, while type A2 fractures were managed with PFNA, InterTan or PFLP. PFNA was the most commonly used fixation material at 95.58%, followed by InterTan at 1.23%, DHS at 1.69%, and PFLP at 1.50% (Figure 1).

### The measurement methods of the TAD and the lateral wall thickness

TAD refers to the distance from the tip of the internal fixation device, such as a screw or a helical blade, to the apex of the femoral head's articular surface. It is essential to identify the apex (the highest point) of the femoral head and the position of the screw tip on the anterior-posterior (AP) and lateral (LAT) x-ray films, respectively, and to measure the distance between these two points. The formula for measuring TAD is as follows: TAD (mm) = [Xap × (Dture/Dap)] + [Xlat × (Dture/Dlat)]. In this equation, Dture represents the actual diameter of the internal fixation, while Dap and Dlat denote the diameter of the internal fixation as observed on the frontal and lateral x-ray film. The variable Xap and Xlat signify the distance from the center of the top of the internal fixation to the apex of the femoral head on the frontal and lateral x-ray film.

The upper boundary of the lateral femoral wall is defined by the lateral femoral muscle ridge, while the lower boundary is marked by the intersection of the lateral femoral cortex and the tangent line of the lower femoral neck. On the anterior-posterior x-ray film, a reference point is established 3 cm below the lateral muscle ridge of the femur. The distance from this reference point to the fracture line, which is inclined upward at an angle of 135°, represents the thickness of the lateral wall.

### Follow-up and evaluation

The patient undergoes regular follow-up appointments at 1, 3, 6, and 12 months post-surgery, during which x-rays of the hip joint are obtained. The patients’ preoperative, postoperative and follow-up imaging data were documented using the Picture Archiving and Communication System (Centricity PACS 4.4). Fracture healing was assessed using two indicators: healing time and healing morphology.

Healing time was categorized into normal healing, delayed healing and non-healing. The criteria for assessing fracture healing include the disappearance of the fracture line on x-ray images, the absence of longitudinal percussion pain in the affected limb during physical examination, and the lack of significant pain during both active and passive movements. The physical examination is conducted by a senior orthopedic physician, while the interpretation of the x-ray images is performed independently by two senior radiologists. The results from both specialists are then integrated to form a comprehensive judgment.

Healing morphology was evaluated based on the difference in neck-shaft angle between the affected and unaffected sides on the x-ray film during the final follow-up, with distinctions made for a difference of ≤5° or >5°. Joint function recovery was assessed using the Oxford scoring system during the final follow-up. This scoring system comprises 12 questions that assess four key aspects: pain (both at rest and during activities), daily activity capability (including tasks such as walking, ascending and descending stairs, and putting on shoes and socks), sleep quality, and the overall impact on life (encompassing social and work-related factors). Each question is rated on a scale from 0 to 4 points, culminating in a maximum total score of 48 points.

Statistical analysis considered various factors: (1) Patient factors such as gender, age, and injury side; (2) Fracture factors including fracture type, lateral wall thickness, and presence or absence of medial wall defects; (3) Internal fixation factors like DHS/PFNA/InterTan/PFLP, main screw position (following the 9-point rule) and tip-apex distance (≤25 mm or >25 mm).

### Statistical analysis

SPSS 24.0 was utilized for data analysis in this study. Measurement data that followed a normal distribution were presented as Mean ± SD and a two independent samples t test was employed to compare groups. Count data were expressed as percentages, and the *x*^2^ test was used for group comparisons. Variables such as gender, age, injury side, AO classification, Evans-Jensen classification, lateral wall thickness, medial support, tip-apex distance and the position of the fixation material on the femoral head were individually analyzed for both the normal healing group and the abnormal healing group. Factor analysis was conducted and factors showing statistically significant differences were further analyzed using Logistics regression. A significance level of *P* < 0.05 was used to determine statistical significance. The odds ratio (OR) is a statistical measure used to represent the ratio of the probability of a certain event occurring in two distinct groups. An OR value of 1 indicates that there is no association between the exposure factor and the outcome. An OR greater than 1 suggests that the exposure may increase the risk of the outcome, while an OR less than 1 indicates that the exposure may reduce the risk of the outcome.

## Results

### Univariate analysis

The results of the univariate analysis indicated statistically significant differences in AO classification, Evans-Jensen classification, apex distance, main nail position and medial support between the two groups (*P* < 0.05). However, gender, age, side and lateral wall thickness did not show statistically significant differences (*P* > 0.05, Table 1).

**Table 1:** Univariate analysis of fracture healing.

Item	Normal healing group (163 cases)	Abnormal healing group (49 cases)	*X*^2^/t value	*P* value
Gender				
Male	77	23	0.052	>0.05
Female	86	26		
Age (Mean ± SD)	78.6 ± 12.8	77.0 ± 8.6	1.297	>0.05
Injury Side				
Left	83	24	0.283	>0.05
Right	80	25		
AO Classification				
A1	46	9	0.653	<0.05
A2	117	40		
Evans-Jensen classification				
I	15	2	2.648	<0.05
II	31	6		
III	58	17		
IV	42	16		
V	17	8		
Lateral wall thickness (mm)				
≤10	72	22	0.180	>0.05
11–20	52	15		
21–30	28	10		
>30	11	2		
Medial support				
Partial defect	59	19	3.826	<0.05
Complete defect	54	23		
No defects	50	7		
Tip-apex distance (mm)				
≤25	89	20	2.487	<0.05
>25	74	29		
Main screw position				
1–3	12	6	2.573	<0.05
4–6	107	19		
7–9	44	24		

### Logistic regression analysis

The results of the Logistic regression analysis indicated that simple fracture type (OR = 1.030), presence of medial wall (OR = 0.395), tip-apex distance ≤25 mm (OR = 0.266) and main screw located in the middle and lower part of the femoral head (OR = 0.986) were significantly associated with normal fracture healing (*P* < 0.05, Table 2). Among all factors, AO type had the greatest influence weight, followed by main screw position; medial support and tip-apex distance had a smaller impact, with medial support having a greater influence than tip-apex distance.

**Table 2:** Logistics regression analysis of fracture healing.

Item	*B*	*SE*	Wald	df	*P* value	*OR*
AO classification	0.030	0.597	2.38	1.00	<0.05	1.030
Medial wall support	−0.928	0.624	12.21	1.00	<0.05	0.395
Tip-apex distance	−1.325	1.026	1.669	1.00	<0.05	0.266
Main screw position	−0.014	0.254	2.36	1.00	<0.05	0.986
Constant	0.366	4.60	12.92	1.00	<0.05	1.422

### Relationship between hip joint function and fracture healing

Based on the postoperative neck-shaft angle and Oxford score criteria, patients with normal fracture healing showed better recovery of hip joint function. The Oxford score for the normal fracture healing group was (42.6 ± 4.5) points, whereas the abnormal healing group scored (35.4 ± 3.2) points. The difference between the two groups was statistically significant (t = 14.8, *P* < 0.05). There were no complications such as infection or vascular and nerve damage reported in any patients. In the abnormal fracture healing group, one patient developed deep vein thrombosis post-surgery but recovered after receiving a filter and thrombolytic treatment. Two patients experienced non-union, which were successfully treated with secondary revision surgery.

## Discussion

Both fracture and treatment factors can significantly impact the outcomes of intertrochanteric fractures. In a study by Hsueh (7), DHS was utilized in treating 1,150 cases, with a postoperative internal fixation cutting rate of 6.8%. Logistic regression analysis identified tip-apex distance as the primary factor influencing efficacy, while internal fixation position, fracture type, reduction quality and patient age also played roles in treatment outcomes. Another study treated 205 elderly cases with DHS and PFNA, reporting a surgical failure rate of 5.9%, Logistic regression analysis indicated that the tip-apex distance greater than 25 mm, severe osteoporosis, unstable fractures and poor reduction could potentially lead to treatment failure (8). Currently, internal fixation is the predominant treatment approach for intertrochanteric fractures, with a lack of quantitative analysis on factors related to intramedullary fixation treatment. Our research highlighted that fracture classification and stability are key factors for fracture healing. Factors such as the presence of the medial wall, maintaining the tip-apex distance less than 25 mm and correct screw position can aid in fracture healing and promote joint function recovery.

### Gender differences in intertrochanteric fractures and treatment choices

Epidemiological surveys have revealed variations in incidence levels among different ages and genders, with women showing a higher incidence rate compared to men (9). In this study, the proportion of female patients was 52.8%, exceeding that of males. The heightened susceptibility of older women to intertrochanteric fractures may be linked to the rapid progression of postmenopausal osteoporosis and a more pronounced decline in local bone quality. Osteoporosis poses significant challenges in fracture treatment. Intramedullary fixation stands out as the preferred treatment approach for intertrochanteric fractures due to its lower trauma and superior stability. Among this patient group, intramedullary fixation constituted approximately 95.58% of all fixation methods. Locking steel plates are not commonly used for treating intertrochanteric fractures, possibly due to design flaws such as high stress concentration at the screw-plate connection, making it susceptible to metal fatigue or fracture (10).

### The impact of patient and fracture factors on the treatment of intertrochanteric fractures

The study indicates that there are no statistically significant differences in gender, age, injury side, and lateral wall thickness between patients in the normal healing group and the abnormal healing group. The impact of age on fracture healing is deemed insignificant. We suggest that this could be attributed to prolonged immobilization and delayed initiation of functional exercises in elderly patients post-surgery. In contrast, younger patients may have a lower tolerance for extended bed rest and are more likely to engage in weight-bearing exercises early on, potentially increasing the risk of delayed fracture healing compared to older patients. Chehade (11) proposed that male patients may have slightly lower tolerance to fractures than female patients, yet there is no significant disparity between the two groups in terms of postoperative functional recovery and walking ability. The lateral wall plays a crucial role in supporting the femoral head and neck, with its thickness influencing the support provided (12). While biomechanical studies suggest that increasing lateral wall thickness enhances support when using DHS, this study found that lateral wall thickness during internal fixation does not significantly impact intertrochanteric fracture healing. This could be due to the main nail of the internal fixation independently supporting the femoral head and neck, with the lateral wall playing a relatively minor role in providing support.

The stability of the fracture is a crucial factor in determining the healing process of intertrochanteric fracture. Chehade (11) conducted a prospective study involving 743 patients, categorizing them into stable and unstable fractures. The research revealed that unstable fractures significantly raised the risk of internal fixation failure, reoperation and mortality rates within one year post-surgery, compared to stable fractures (13). Moreover, our study delved into three distinct fracture factors—fracture type, lateral wall thickness, and medial support—for analysis, shedding light on the correlation between increased comminution and instability in AO type A1 and A2 fractures. The Evans-Jensen classification, primarily focused on fracture location rather than stability, was deemed less relevant in predicting fracture healing outcomes. Notably, medial support emerged as a critical postoperative stability determinant for intertrochanteric fractures, with the posteromedial bone fragment, including the femoral calcar, playing a key role in resisting varus stress on the femoral neck (14). However, the challenge of exposing, reducing, and fixing posteromedial bone fragments underscores the importance of restoring medial support through reestablishing contact with anteromedial bone fragments as a fundamental principle in current intertrochanteric fracture treatment.

### Relationship between surgical factors and fracture efficacy

The positioning of internal fixation in the femoral neck is a crucial factor in the treatment of intertrochanteric fractures. One of the most notable methods for assessing the location of internal fixation in the femoral head is the nine-square grid partition method introduced by Cleveland (15) in 1959. This method divides the midline of the femoral head into nine squares, providing a framework to describe the placement of screws in the femoral head and the relative positioning of bones. Biomechanical research has demonstrated that placing screws beneath the femoral head, regardless of using internal or external fixation, can significantly enhance axial stiffness and torsional strength of the fixation, while reducing stress concentration on the internal fixation and bone tissue, thereby promoting fracture healing (16, 17). Findings from our study indicate a lower risk of fracture healing when the main screw is positioned in the middle or lower part of the femoral head, aligning with previous studies. However, it is important to note that the nine-square grid partitioning method offers a general analysis of screw placement and does not provide precise information on the depth of internal fixation. Therefore, a more objective parameter is necessary to accurately describe the exact location of internal fixation.

The concept of tip-apex distance was first proposed by Baumgaertner (18) in 1995. This distance is measured in millimeters by summing the distances from the tip of the screw on x-rays taken immediately after surgery to specific points on the femoral head. A tip-apex distance of ≤25 mm has been widely accepted by orthopedic surgeons as effective in preventing screw cutout. However, there is ongoing debate regarding the optimal range for tip-apex distance in intramedullary fixation (19). Observations from patient treatments in our study suggest that a tip-apex distance of ≤25 mm promotes normal fracture healing, aligning with the effective range of DHS. On the other hand, a tip-apex distance that is too small can lead to screw cutout. For instance, a study by Nikoloski (20) on patients treated with PFNA revealed that those with tip-apex distance <20 mm experienced cut out of the femoral head, while those with >20 mm did not. This highlights the importance of controlling tip-apex distance within a specific range during PFNA fixation. It is recommended that the main screw be positioned in the middle to lower part of the femoral head using three-dimensional navigation, with a strict control of the TAD to remain within ≤25 mm. Further biomechanical research is necessary to determine the appropriate range of tip-apex distance for intramedullary fixation and its impact on fracture healing and screw retention.

### The relationship between fracture healing and joint function recovery

Fracture healing is a crucial factor in enabling rehabilitation and facilitating functional recovery. We examined the correlation between fracture healing and joint function recovery, finding that patients with normal fracture healing experienced better joint function, demonstrating a consistent relationship between the two (3, 21). Two cases of non-healing fractures were discussed in this study. In one case, an AO type A2.3 fracture was fixed with PFNA, but due to incomplete reduction of fracture displacement and the use of a short spiral blade, cutting occurred after the surgery, leading to nonunion of the fracture. In the other case, PFLP fixation was employed, and the patient engaged in early weight-bearing activities post-operation, resulting in nonunion of the fracture and internal fixation failure, necessitating a second revision surgery.

## Conclusion

Fracture type and the stability of internal fixation are crucial factors influencing fracture healing. In internal fixation, securing the main screw at the middle and lower portion of the femoral head, while maintaining a tip-apex distance of ≤25 mm, has been shown to promote fracture healing. Given the retrospective study, there is a potential for bias in case selection. Gathering more cases and refining classification criteria could lead to more meaningful results and better guide fracture treatment and rehabilitation.

## Data Availability

The raw data supporting the conclusions of this article will be made available by the authors, without undue reservation.
